# Is Friedreich ataxia an epigenetic disorder?

**DOI:** 10.1186/1868-7083-4-2

**Published:** 2012-01-30

**Authors:** Daman Kumari, Karen Usdin

**Affiliations:** 1 Section on Gene Structure and Disease, Laboratory of Cell and Molecular Biology, National Institute of Diabetes, Digestive and Kidney Diseases, National Institutes of Health, Bethesda, MD 20892-0830, USA

**Keywords:** Friedreich ataxia, heterochromatin, histone modifications, transcription, splicing, triplex

## Abstract

Friedreich ataxia (FRDA) is a debilitating and frequently fatal neurological disorder that is recessively inherited. It belongs to the group of genetic disorders known as the Repeat Expansion Diseases, in which pathology arises from the deleterious consequences of the inheritance of a tandem repeat array whose repeat number exceeds a critical threshold. In the case of FRDA, the repeat unit is the triplet GAA•TTC and the tandem array is located in the first intron of the frataxin (*FXN*) gene. Pathology arises because expanded alleles make lower than normal levels of mature *FXN *mRNA and thus reduced levels of frataxin, the *FXN *gene product. The repeats form a variety of unusual DNA structures that have the potential to affect gene expression in a number of ways. For example, triplex formation *in vitro *and in bacteria leads to the formation of persistent RNA:DNA hybrids that block transcription. In addition, these repeats have been shown to affect splicing in model systems. More recently, it has been shown that the region flanking the repeats in the *FXN *gene is enriched for epigenetic marks characteristic of transcriptionally repressed regions of the genome. However, exactly how repeats in an intron cause the *FXN *mRNA deficit in FRDA has been the subject of much debate. Identifying the mechanism or mechanisms responsible for the *FXN *mRNA deficit in FRDA is important for the development of treatments for this currently incurable disorder. This review discusses evidence for and against different models for the repeat-mediated mRNA deficit.

## Introduction

Friedreich ataxia (FRDA) (OMIM 229300; http://www.omim.org/entry/229300), first described in 1863 by Nikolaus Friedreich, is a relentlessly progressive disorder caused by mutations in the frataxin (*FXN) *gene. It is the most common heritable ataxia in Caucasians [1]. The major pathological changes include loss of myelinated axons in peripheral neurons, particularly in the dorsal root ganglia, the degeneration of posterior columns of the spinal cord and the loss of peripheral sensory nerve fibers. Myocardial muscle fibers also degenerate and are replaced by macrophages and fibroblasts. The net result of these and other changes include not only limb and gait abnormalities, but also hypertrophic cardiomyopathy, limb muscle weakness, absent lower limb reflexes and a positive extensor plantar response (Babinski sign). Decreased vibration sense, skeletal abnormalities, dysarthria, and diabetes are common comorbid features. Many symptoms become apparent during adolescence. Loss of ambulation occurs roughly 15 years after disease onset with > 95% of patients becoming wheelchair bound by the age of 45. Early mortality due primarily to cardiac failure is not uncommon [2,3].

### The most common FRDA mutation is an expansion of the GAA•TTC repeat tract in intron 1 of the frataxin gene

**Figure 1:**
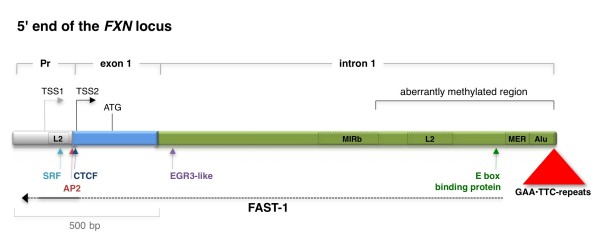
**The 5' end of the frataxin (*FXN*) gene showing the minimal promoter, exon 1 and the promoter proximal end of intron 1**. TSS1 and TSS2 refer to transcription start sites described in two different studies. TSS1 was identified based on a cDNA clone isolated from cardiac mRNA [4]. TSS2 is the major start site in lymphoblastoid cells identified by primer extension [52]. The positions of various interspersed repeated sequences are indicated by the rectangles outlined with black dashed lines. The dotted black arrow indicates the estimated extent of the FXN antisense transcript-1 (FAST-1) transcript based on nested PCR [25]. The solid black line superimposed on it is the region that could be amplified by a single round of PCR. The larger antisense transcript includes an open reading frame (ORF) with a non-canonical Kozak sequence whose significance is unknown. The ORF is intact in humans but truncated in closely related primates. Arrows indicate the location of the binding sites for serum response factor (SRF), activator protein 2 (AP2) [73], CCCTC-binding factor (CTCF) [25], an early growth response protein 3 (EGR3)-like factor [73] and an E-box binding protein [48] which have been shown to be positive regulators of *FXN *expression.

FRDA is inherited in an autosomal recessive fashion. The affected gene, frataxin (*FXN*) (OMIM 606829; http://omim.org/entry/606829), is located on chromosome 9q13 in humans [4]. The first intron contains a GAA•TTC repeat tract embedded in the central poly(A) tract of an AluSq element from which it probably arose [5]. The GAA•TTC repeat tract, which is located approximately 1.3 kb downstream of the major *FXN *transcription start site (TSS), is polymorphic in the human population (Figure 1). While normal alleles have between 8 to 33 repeats, most individuals with FRDA have 2 *FXN *alleles each with > 90 repeats, the majority having 600 to 900 repeats [4]. A minority of patients (approximately 4%) are compound heterozygotes, having one allele with > 90 repeats and a second allele with a small deletion or point mutation in the *FXN *open reading frame. No cases of individuals with deletions or point mutations in both alleles are known [4].

Since most FRDA patients have at least one allele that contains a large repeat expansion, FRDA is considered to belong to a group of approximately 20 human genetic disorders known as the Repeat Expansion Diseases. In this group of diseases pathology arises from the consequences of inheritance of alleles with repeat numbers above a critical pathological threshold, which in the case of FRDA is approximately 90 repeats. The basis of the underlying expansion mutation responsible for these disorders is unknown, and problems with DNA replication, recombination and repair have all been suggested as possible mechanisms [6].

### FRDA results from a deficiency of *FXN *mRNA

Expansion results in *FXN *mRNA levels that are 4% to 29% of normal [7]. There is an inverse relationship between repeat number and the amount of *FXN *mRNA produced. The *FXN *gene product, frataxin, is a small, highly conserved, acidic protein that is essential for life [8]. It is highly expressed in the dorsal root ganglia, the granular layer of the cerebellum as well as the heart, pancreas, thymus, brown fat, muscle and liver. Although the protein is nuclear encoded, it functions in the mitochondria where it is thought to be involved in the biosynthesis of iron-sulfur clusters (ISCs) [9], the complexes that serve as prosthetic groups for a variety of enzymes involved in energy and iron metabolism, purine synthesis and DNA repair. However, its precise role is currently unknown.

In principle, an *FXN *mRNA deficit could arise via an effect of the intronic repeats on the efficiency of transcription or some post-transcriptional event. However, no difference has been seen in the decay rate for the mature transcripts produced from normal and FRDA alleles [10]. Thus, the *FXN *mRNA deficit presumably results from events occurring at the level of transcription, and/or pre-mRNA stability or processing.

### The GAA•TTC repeats form an intrinsic block to transcription elongation in simple model systems

**Figure 2:**
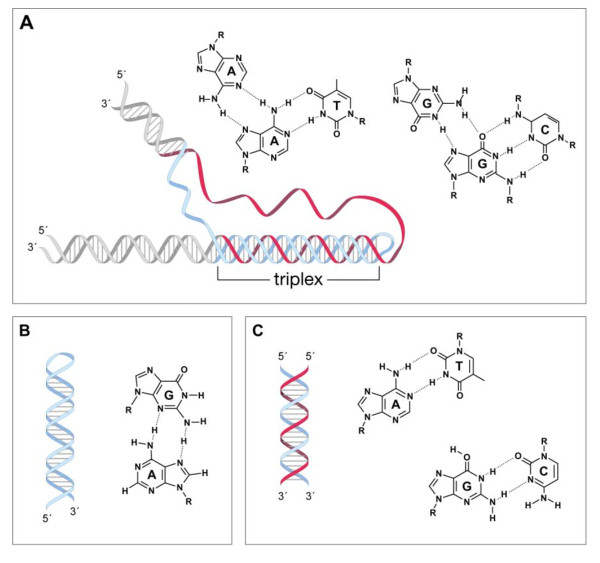
**Examples of different structures formed by GAA•TTC repeats showing their constituent hydrogen bonding schemes**. **(A) **A purine:purine:pyrimidine triplex [11,12]. This triplex involves Hoogsteen hydrogen bonding between a purine already in a Watson-Crick base pair with a pyrimidine, and a purine from a different region of the repeat. **(B) **A GAA hairpin [61]. Various hydrogen bonding schemes involving G•G, G•A and A•A base pairs have been proposed [74] including the one shown. However, the precise molecular details of the GAA hairpin are unknown. **(C) **A parallel duplex in which, unlike the antiparallel configuration of normal Watson-Crick duplex, the polarity of the two base-paired strands is the same [75]. In this configuration the base pairs involve a reverse Watson-Crick orientation and two, rather than three, hydrogen bonds.

*In vitro *transcription of templates containing as few as 11 GAA•TTC repeats produces less full-length RNA than templates with no repeats [11]. The repeats form a variety of unusual secondary structures under the same conditions (Figure 2). These structures include purine:purine:pyrimidine and pyrimidine:purine:pyrimidine triplexes [11-15] and a related structure known as sticky DNA [16]. It has been suggested that triplex formation could affect transcription by sequestering transcription factors or RNA polymerase (RNAP) [17,18]. It has also been suggested that a pre-existing triplex or sticky DNA blocks RNAP by making it more difficult for the transcription complex to unwind the template [17].

However, whether the steady state levels of negative superhelicity present in mammalian chromosomes are high enough to allow the formation of such structures is unclear. β-Alanine-linked pyrrole-imidazole polyamides have been shown to bind GAA•TTC tracts with high affinity, to block sticky DNA formation and to increase *FXN *expression in cells from individuals with FRDA [19]. This would be consistent with a role for sticky DNA in FRDA. However, the specificity of these polyamides is uncertain and thus, the molecular basis of their effect is unclear.

**Figure 3:**
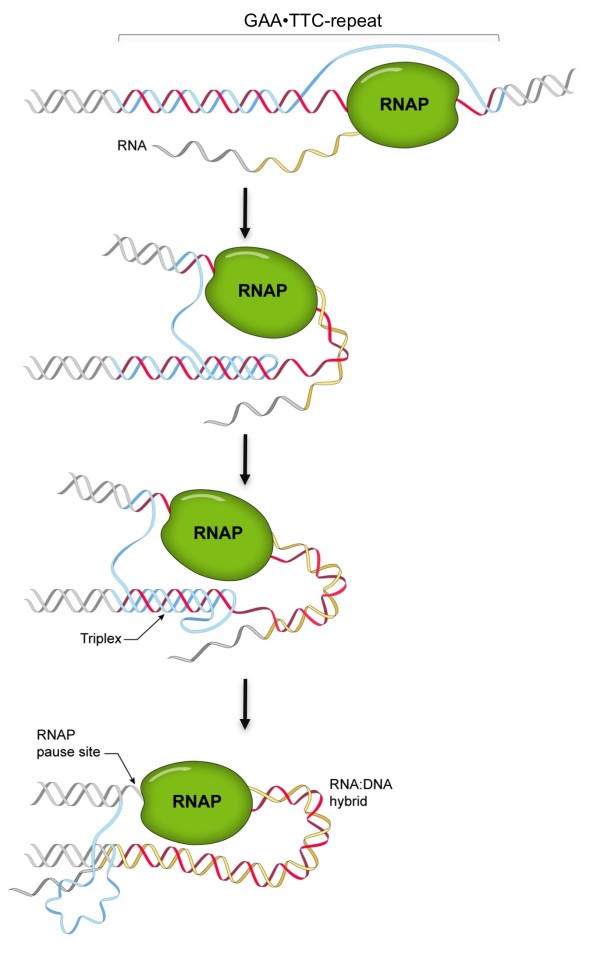
**A triplex/RNA:DNA hybrid model for Friedreich ataxia (FRDA) **[11,12,20]. Transcription through the repeat leaves the non-template purine-rich strand transiently unpaired. This strand can then fold back and interact with the duplex that has already reannealed behind the RNA polymerase (RNAP), thereby forming a triplex. Triplex formation, in turn, leaves the pyrimidine-rich strand in the second half of the repeat free to form a hybrid with the nascent RNA. This may be facilitated by the particular stability of an RNA:DNA hybrid containing a purine-rich RNA strand [76]. Evidence suggests that nucleation of this hybrid leads to unwinding of the triplex and the formation of a long persistent RNA:DNA hybrid that involves the whole repeat. The net result is the formation of a stable R loop in which the pyrimidine strand of the repeat is hybridized to the nascent transcript leaving the purine-rich strand unpaired. The RNAP becomes trapped on the template at the 3' end of the repeat.

In addition to preformed triplexes, there is also evidence to suggest that triplexes formed during transcription *in vitro *lead to the formation of an RNA:DNA hybrid as illustrated in Figure 3[11,12,20]. This results in a block to transcription and trapping of RNAP on the template at the end of the repeat. Other sequences or conditions that favor the formation of R-loops also impede transcription [21,22]. Thus it is reasonable to think that an R-loop on a FRDA allele, however it is formed, could cause a block to transcription elongation. Single-stranded nicks in the template, perhaps arising from attempts to repair one of the structures formed by the repeat, can also increase the likelihood that R-loops will form during subsequent rounds of transcription [23]. Furthermore, work *in vitro *suggests that R-loops could also arise via bidirectional transcription through the repeat [24]. An antisense transcript, FXN antisense transcript-1 (FAST-1), has been identified in the *FXN *gene that could potentially contribute to such hybrids (see Figure 1). However, its 5' end has not been mapped, its concentration is low and it is unclear at this time whether it includes the repeat [25].

While direct proof of the formation of an R-loop by the FRDA GAA•TTC repeats in mammalian cells is lacking, other purine-rich repeats are known to do so [26]. In addition, the promoter distal end of the repeat in human induced pluripotent cells generated from patient cells is known to bind the mismatch repair proteins MSH2 and MSH3 [27], which would be consistent with the formation of an unusual DNA conformation of some sort at this locus.

While a consistent inhibition of transcription elongation is seen with different RNAPs on naked DNA templates *in vitro *[11,12,16,28,29], conflicting results have been seen with mammalian nuclear extracts and episomes in yeast and mammalian cells [16,28,30-32]. Integrated constructs do show a consistent mRNA decrease in cells and mouse models [33-35], but whether this effect is mediated at the level of transcription elongation is unclear.

### The GAA•TTC repeats affect splicing in model systems

**Figure 4:**
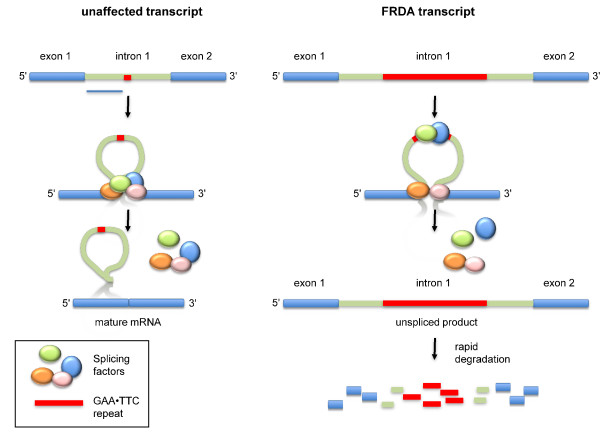
**Altered splicing model for Friedreich ataxia (FRDA) **[30]. On unaffected frataxin (*FXN*) alleles the repeat is too short to significantly impact splicing. However, once the repeat number exceeds 90, splicing factors that are normally involved in the proper splicing of the *FXN *gene become mislocalized such that normal splicing is prevented. This could be the result of binding of splicing factors to the repeat, preventing their normal assembly at the splice junctions. Alternatively, the unrestrained spread of these or other repeat-binding proteins, such as heterogeneous nuclear ribonucleoprotein (hnRNP) A1 [77], could block access of factors needed for proper splicing analogous to what has been proposed for HIV [78]. It may also be that the repeat sequesters serine/arginine (SR) proteins such as alternative splicing factor/splicing factor 2 (ASF/SF2). Since these proteins are is required for 5' splice site selection and cleavage [79], this could lead to a local deficiency at the splice site and thus the failure to efficiently remove intron 1.

A so-called frataxin minigene construct, containing a CMV promoter, the *FXN *exon 1, part of intron 1 and all of exon 2, that was transfected into mammalian cells, showed a decreased splicing efficiency when the GAA-rich strand was transcribed but not the TTC-strand [30]. It was thus suggested that the deficit of mature *FXN *mRNA in FRDA results from aberrant mRNA splicing in which intron 1 is retained. The aberrant splicing seen with the minigene was attributed to the ability of the repeats to bind splicing factors such as the serine/arginine (SR)-rich protein family and the proteins heterogeneous nuclear ribonucleoprotein (hnRNP) A1 and hnRNP A2 as outlined in Figure 4, although how binding of these factors would lead to intron retention is unclear.

However, since total *FXN *mRNA abundance is reduced in FRDA cells, any mis-spliced transcript would have to be rapidly degraded for aberrant splicing to account for the mRNA deficit, which was not the case with the minigene tested [30]. In addition, the splicing abnormalities in the frataxin minigene were context and position dependent. This is important since in this construct both the repeat context and position differed from what is seen in the *FXN *locus.

The FRDA GAA•TTC repeats have also been shown to reduce splicing in yeast [36]. This effect was attributed to the increased length of the intron rather than any specific effects of the repeat *per se*. In yeast the largest known intron is < 1 Kb and in these organisms splicing efficiency is related to intron length [37]. However, many efficiently spliced human introns are much longer, with the human genome containing > 3000 genes with introns > 50 Kb. Since the *FXN *intron 1 of normal alleles is already 11 Kb and cases of FRDA are apparent with as few as 90 repeats, it seems unlikely that a change in intron length *per se*, is responsible for the reduced *FXN *expression in FRDA.

Furthermore, studies of transcripts produced from the intact *FXN *gene did not detect any splicing abnormalities in FRDA cells [10,28]. However, since the existence of a very unstable splice isoform is difficult to definitively exclude, this issue is still unresolved.

### Expansion of the FRDA GAA•TTC repeat tract also causes epigenetic changes

While it has been known for some time that a subset of Repeat Expansion Diseases are associated with heterochromatin formation, notably those disorders arising from CGG•CCG repeat expansion such as fragile X syndrome (FXS) [38], the idea that the FRDA GAA•TTC repeats produce aberrant epigenetic modifications has only recently been appreciated. In part, the possibility that FRDA could be an epigenetic disorder was not initially entertained since unlike the affected gene in FXS, significant transcription still occurs from most FRDA alleles and early thinking in the field was that DNA methylation was required for epigenetic silencing [39-43]. Since the FRDA repeat contains no CpG residues, the only dinucleotide subject to significant methylation in mammals, non-epigenetic mechanisms, like those described earlier, initially received more attention.

However, it is now appreciated that even in those repeat expansion diseases where the repeat has a high density of CpG residues, such as FXS, DNA methylation is probably not the first step in heterochromatinization [44,45]. Furthermore, the expanded CTG•CAG repeats in myotonic dystrophy type 1 (DM1) are associated with heterochromatin despite their lack of CpG residues [46]. In addition, work with transgenic mice containing GAA•TTC repeats or CAG•CTG repeats showed that the repeats conferred variegation in the expression of a linked transgene, analogous to position effect variegation (PEV) in *Drosophila *[47]. These observations suggested that, despite the absence of methylatable residues, the FRDA repeats might trigger the formation of heterochromatin that could spread to adjacent sequences.

While the repeat itself cannot be methylated, DNA methylation could potentially occur secondarily to other chromatin changes in the region flanking the repeat. Consistent with that idea, we have shown that while DNA methylation is seen in the region flanking the repeat on normal alleles, perhaps due to spreading from adjacent Alu elements, more extensive DNA methylation is seen in this region in patient cells [33,48]. A direct relationship between repeat length and the extent of DNA methylation has also been found in patient cells [49]. Since disease severity is related to repeat length, a direct relationship between disease severity and DNA methylation thus also exists.

Not only is DNA methylation more extensive on FRDA alleles, but the methylation protection of 3 CpG residues that is seen upstream of the repeat on unaffected alleles is also lost [48]. One of these residues is within an E-box site that is important for maximal promoter activity in reporter assays in mouse myoblast cells. However, plasmids that are specifically methylated at this site do not show reduced transcription [48]. This suggests that loss of factor binding does not occur secondarily to DNA methylation, but rather that protein binding normally protects those CpG residues from methylation. Thus, the loss of the normal methylation 'footprint' in FRDA cells likely reflects chromatin changes that restrict access of these factors to their normal binding sites. Consistent with this view, FRDA patient alleles have been shown to be enriched for a variety of histone modifications characteristic of silenced genes including hypoacetylated H3 and H4 and dimethylation and trimethylation of histone H3 lysine 9 (H3K9) [48,50]. These histone modifications are highest in the regions flanking the repeat [50-52].

Aberrant DNA methylation does not extend as far as the promoter in any of the patient cell lines that have been tested thus far. However, whether histone modifications extend into the promoter is still controversial. The wide variation in the level of histone modifications seen in normal cells, the use of FRDA cell lines with very different repeat numbers and mRNA levels and differences in the experimental design and data analysis have added to the difficulty in reaching a consensus.

However, to date there have been a number of reports of a histone profile typical of transcriptionally repressed genes on the affected *FXN *promoter in lymphoblastoid cells [25,52], the brains of affected individuals [33] and in a cell culture model [35]. Enrichment of repressive chromatin marks on the *FXN *promoter has also been reported in the brain and heart in transgenic mice models of the disorder [33]. In addition, enrichment of the α and γ isoforms of heterochromatin protein 1 (HP1), a non-chromosomal protein associated with heterochromatin, on the promoter and the loss of CCCTC-binding factor (CTCF) binding to the promoter region in patient cells lends support to the idea that epigenetic changes originating in the repeat can spread to the 5' end of the *FXN *gene [25].

### What is the basis of the epigenetic changes?

It has been suggested that the loss of CTCF binding is responsible for the observed histone changes on FRDA alleles [25]. However, this raises the question of what leads to the loss of CTCF binding. Since heterochromatin can be generated by the repeats embedded in a completely different sequence context [35,47] and levels of the repressive histone modifications are highest in the region of the *FXN *gene that includes the repeat [50-52], it may be that the trigger for these epigenetic modifications is specifically related to some intrinsic property of the repeat itself as has been suggested for FXS [53]. This effect may be at the DNA level perhaps via the ability of the repeat to bind proteins that then recruit silencing factors [54]. It could also be a consequence of the repair of DNA damage occurring in the repeat [55-57]. An unusual structure formed by the FRDA repeat may contribute to this process if it were trigger the DNA damage response. The binding of MSH2/MSH3 complexes to the region containing the repeat in patient cells lends weight to the idea that some sort of structure formed by the repeat is recognized by the cell as a site of DNA damage [27]. It is also possible that reduced transcription, resulting perhaps from a triplex/RNA:DNA hybrid formation, leads to heterochromatic changes, as it does in some plant genes by favoring the recruitment of H3K27 trimethylation (H3K27Me3) [58].

It could also be that heterochromatinization is RNA dependent perhaps involving a long non-coding RNA (lncRNA), as has been described for HOX genes and the lncRNA HOTAIR [59]. The non-coding RNA could be generated *in cis *or *trans*. Recent work has shown that the formation of a DNA:RNA triplex between a chromosomally located gene and ectopic RNA leads to enrichment of the DNA with H4K20Me3 and subsequent gene silencing [60]. Formation of such a triplex by the GAA•TTC repeat and either the repeat region in the sense or antisense transcript could thus lead to heterochromatin initiation within the repeat. Alternatively, if RNA containing a large number of GAA repeats can form hairpin-like GAA repeats in DNA [61], they may be source of double-stranded (ds)RNA for the RNA interference (RNAi) pathway. Transcripts containing the repeats may thus enter the RNAi pathway as has been demonstrated for the repeats responsible for FXS and DM1 [62,63].

### How could these chromatin changes affect *FXN *transcription?

Since the repeat-associated chromatin changes are located in both the transcriptional unit and in the promoter of at least some patient cells, they have, in principle, the potential to affect expression of the *FXN *gene in a number of different ways. This effect could be exerted close to the start of transcription mediated by chromatin changes on the promoter. In addition, CTCF binding has been shown to play an important role in *FXN *expression [25]. So, simply the loss of this factor from patient alleles could lead to reduced rates of transcription. Furthermore, even in the absence of altered promoter chromatin, histone and DNA methylation changes in the intron that lead to loss of binding of important regulatory factors may affect transcription initiation or early steps in elongation. Since DNA methylation in the body of a gene can affect the efficiency of transcription elongation [64], an effect on transcription through the intron is also possible.

### Do epigenetic changes account for the *FXN *mRNA deficit?

The role of chromatin changes in causing the *FXN *mRNA deficit in FRDA is currently the subject of much debate. Histone deacetylase inhibitors have been shown to increase *FXN *expression in FRDA primary lymphocytes and the brain and heart of a knock-in mouse model of the disorder [34,50]. The histone deacetylase, HDAC3, has been identified as an important target of these drugs [65]. The increase in *FXN *expression is accompanied by an increase in histone acetylation on FRDA alleles. However, it has been reported that while the histone methyltransferase inhibitor BIX-01294 reduced the levels of H3K9 dimethylation and trimethylation on FRDA alleles, no accompanying increase in *FXN *transcription was seen. This has led to the suggestion that epigenetic changes are not responsible for the *FXN *deficit and that repeat expansion causes FRDA by forming a structural block to transcription elongation [10].

This idea would appear to be supported by the observation that phosphorylation of serine 5, a mark characteristic of the initiating form of RNA polymerase II (Pol II Ser5-P), is present at similar levels at TSS1 [10], a transcription start site identified in early studies [4]. However, recent work has shown that the major TSS (TSS2) used in lymphoblastoid cells, the cell type used for these studies, is closer to the start of the *FXN *open reading frame than previously thought [52]. This is relevant since the initiating form of Pol II is typically found to have a narrow distribution at or downstream of the TSS [66]. When a region immediately downstream of TSS2 was examined, reduced levels of the initiating form of Pol II [52] as well as total Pol II [51] were seen in FRDA patient cells. A reduced level of H3K4 trimethylation (H3K4Me3) was also seen the region in the region immediately downstream of TSS2 in patient cells [52]. Deposition of this histone mark occurs early in the transcription cycle primarily on the first nucleosome [67,68]. Trimethylation of H3K4 is thought to be required for both recruitment of the basal transcription machinery and for transcription initiation on genes that, like *FXN*, lack a TATA box [69]. In other genes, deposition of this histone mark is thought to occur immediately downstream of the promoter in a manner dependent on the levels of the initiating form of Pol II [69,70]. In either event, the reduced level of H3K4Me3 seen on patient alleles suggests that a problem with transcription from FRDA templates is apparent very early in the transcription cycle, perhaps at the level of polymerase recruitment or transcription initiation.

More recently it has been suggested that the reduced levels of Pol II are not due to reduced initiation but to reduced promoter proximal pausing [51]. This conclusion was based on the fact that no difference was seen in H3K4Me3 levels on unaffected and affected alleles at the 5' end of the gene. However, in this study the region examined was upstream of what we now know to be the major TSS, in a part of the promoter that also did not show differences between affected and unaffected alleles in earlier reports [10,52]. Since H3K4Me3 is highest on nucleosomes immediately downstream of the TSS, the lower levels of H3K4Me3 that were seen on patient alleles just upstream of the repeat in the study of Kim *et al. *[51], in fact lend support to the idea that early events in transcription occurring prior to or during H3K4 trimethylation are abnormal in FRDA. However, further work is needed to establish precisely what step or steps are affected.

Whatever the cause of the reduced levels of Pol II on FRDA alleles, the lower levels of H3K36 trimethylation, a histone mark associated with transcription elongation, in the promoter proximal region [10,51,52], supports the idea that there is an effect of the repeat on transcription very close to the TSS more than 1 kb upstream of the repeat. Furthermore, the reduced levels of H3K79Me2, another mark of transcription elongation, found upstream of the repeat in patient cells [51], further strengthens the idea that there is reduced transcription in the region preceding the repeat.

This is not to say that there is not a problem with transcription closer to the repeat as well. An additional effect of repeat expansion on Pol II elongation is suggested by the reduced accumulation of H3K36Me3 downstream of the repeat on FRDA alleles [10,51,52]. Whether this represents an effect of the histone changes and DNA hypermethylation in the vicinity of the repeat in patient cells or a chromatin-independent process remains to be seen.

The relationship between GAA repeat number and the extent of intron DNA methylation raises the possibility that the epigenetic changes on smaller alleles may be smaller than on larger alleles and less likely to extend into the promoter. Thus the relative contribution of promoter-proximal and promoter-distal events may vary with repeat number.

## Conclusions

**Figure 5:**
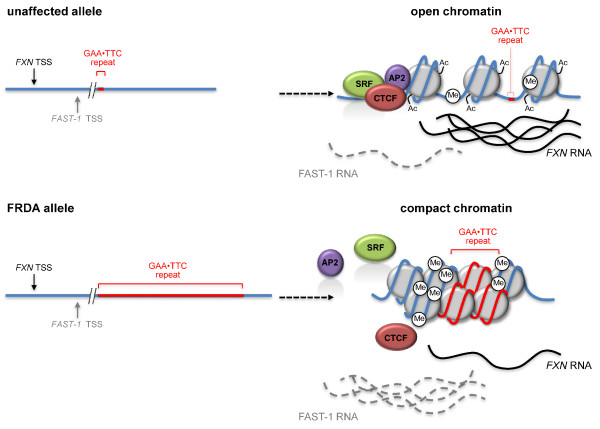
**Diagrammatic representation of an epigenetic model for Friedreich ataxia (FRDA)**. Not shown to scale. Unaffected alleles are aberrantly methylated in the region flanking the repeat. Nonetheless, the 5' end of the gene is associated with histones that are enriched for marks of active chromatin. In particular, acetylation of histone H3 and H4 is high. The net result is that the chromatin is open and permissive for transcription. Transcription factors including serum response factor (SRF), activator protein 2 (AP2) [73] and CCCTC-binding factor (CTCF) [25] associate with the 5' end of the gene. An early growth response protein 3 (EGR3)-like factor binds to the 5' end of intron 1 [73] and an E-box binding protein [48] bind to the region immediately upstream of the repeat. Under these conditions transcription initiation and elongation takes place normally. In contrast, FRDA alleles become associated with histones that are hypoacetylated and show more extensive DNA methylation in the region flanking the repeat. The net effect of these and other histone changes is the formation of a compact chromatin configuration. This reduces binding of transcription factors and both frataxin (*FXN*) transcription initiation and elongation are reduced. Loss of CTCF binding is correlated with an increase in the amount of FXN antisense transcript-1 (FAST-1) RNA that is transcribed antisense to *FXN*, but how this relates to silencing is unclear. TSS: transcription start site.

An effect of the GAA•TTC repeat on events occurring > 1 kb away at the *FXN *promoter is difficult to reconcile with an effect of aberrant splicing. It is also difficult to reconcile with a direct effect of the formation of a triplex/R-loop unless problems occurring in the repeat lead to the buildup of stalled polymerases that stretches back to the promoter. Therefore, perhaps the most likely explanation for the promoter proximal effects is that the repeat-mediated epigenetic changes generate a chromatin configuration that is less permissive for early steps in transcription as illustrated in Figure 5. That is that FRDA is, at least in part, a disorder of epigenetic dysregulation. The lack of an effect of BIX-01294 on *FXN *mRNA yield can be reconciled with this idea, if histone marks other than H3K9 methylation need to be removed before a chromatin conformation permissive for transcription is reestablished, as has been suggested for a number of other repressed genes [71,72]. If this is the case, it would suggest that histone deacetylase inhibitors, which are currently in clinical trials for treating FRDA, are probably acting on one of the direct causes of the transcription deficit. Such a mechanism would not necessarily preclude a role for triplexes/R-loops in events occurring at the promoter if, as discussed earlier, such structures contribute in some way to the formation of heterochromatin.

Whether problems with Pol II elongation in the vicinity of the repeat are epigenetically mediated or arise from a physical block to elongation like that formed by triplex/R-loops also remains an open question, with some data supporting a role for chromatin-mediated events and some data favoring a chromatin-independent mechanism. It may be that both mechanisms contribute to the *FXN *mRNA deficit in some way and further work will be necessary to understand the relative contribution of these mechanisms to the *FXN *mRNA deficit responsible for FRDA.

## Competing interests

This work was made possible by a grant to KU from the Intramural Program of NIDDK (DK057810). The Authors declare that there is no conflict of interest.

## Authors' contributions

Both DK and KU contributed equally to the writing of this manuscript. Both authors read and approved the final manuscript.

## References

[B1] Cossee M Schmitt M Campuzano V Reutenauer L Moutou C Mandel JL Koenig M Evolution of the Friedreich's ataxia trinucleotide repeat expansion: founder effect and premutations Proc Natl Acad Sci USA 1997 94 7452 7457 10.1073/pnas.94.14.7452 9207112 PMC23842

[B2] Harding AE Early onset cerebellar ataxia with retained tendon reflexes: a clinical and genetic study of a disorder distinct from Friedreich's ataxia J Neurol Neurosurg Psychiatry 1981 44 503 508 10.1136/jnnp.44.6.503 7276963 PMC491030

[B3] Harding AE Zilkha KJ 'Pseudo-dominant' inheritance in Friedreich's ataxia J Med Genet 1981 18 285 287 10.1136/jmg.18.4.285 7277422 PMC1048733

[B4] Campuzano V Montermini L Moltò MD Pianese L Cossée M Cavalcanti F Monros E Rodius F Duclos F Monticelli A Zara F Cañizares J Koutnikova H Bidichandani SI Gellera C Brice A Trouillas P De Michele G Filla A De Frutos R Palau F Patel PI Di Donato S Mandel JL Cocozza S Koenig M Pandolfo M Friedreich's ataxia: autosomal recessive disease caused by an intronic GAA triplet repeat expansion Science 1996 271 1423 1427 10.1126/science.271.5254.1423 8596916

[B5] Clark RM Dalgliesh GL Endres D Gomez M Taylor J Bidichandani SI Expansion of GAA triplet repeats in the human genome: unique origin of the FRDA mutation at the center of an Alu Genomics 2004 83 373 383 10.1016/j.ygeno.2003.09.001 14962663

[B6] Pearson CE Nichol Edamura K Cleary JD Repeat instability: mechanisms of dynamic mutations Nat Rev Genet 2005 6 729 742 10.1038/nrg1689 16205713

[B7] Campuzano V Montermini L Lutz Y Cova L Hindelang C Jiralerspong S Trottier Y Kish SJ Faucheux B Trouillas P Authier FJ Dürr A Mandel JL Vescovi A Pandolfo M Koenig M Frataxin is reduced in Friedreich ataxia patients and is associated with mitochondrial membranes Hum Mol Genet 1997 6 1771 1780 10.1093/hmg/6.11.1771 9302253

[B8] Cossee M Puccio H Gansmuller A Koutnikova H Dierich A LeMeur M Fischbeck K Dolle P Koenig M Inactivation of the Friedreich ataxia mouse gene leads to early embryonic lethality without iron accumulation Hum Mol Genet 2000 9 1219 1226 10.1093/hmg/9.8.1219 10767347

[B9] Rotig A de Lonlay P Chretien D Foury F Koenig M Sidi D Munnich A Rustin P Aconitase and mitochondrial iron-sulphur protein deficiency in Friedreich ataxia Nat Genet 1997 17 215 217 10.1038/ng1097-215 9326946

[B10] Punga T Buhler M Long intronic GAA repeats causing Friedreich ataxia impede transcription elongation EMBO Mol Med 2010 2 120 129 10.1002/emmm.201000064 20373285 PMC3377279

[B11] Grabczyk E Usdin K The GAA*TTC triplet repeat expanded in Friedreich's ataxia impedes transcription elongation by T7 RNA polymerase in a length and supercoil dependent manner Nucleic Acids Res 2000 28 2815 2822 10.1093/nar/28.14.2815 10908340 PMC102661

[B12] Grabczyk E Usdin K Alleviating transcript insufficiency caused by Friedreich's ataxia triplet repeats Nucleic Acids Res 2000 28 4930 4937 10.1093/nar/28.24.4930 11121484 PMC115239

[B13] Jain A Rajeswari MR Ahmed F Formation and thermodynamic stability of intermolecular (R*R*Y) DNA triplex in GAA/TTC repeats associated with Freidreich's ataxia J Biomol Struct Dyn 2002 19 691 699 11843630 10.1080/07391102.2002.10506775

[B14] Mariappan SV Catasti P Silks LA Bradbury EM Gupta G The high-resolution structure of the triplex formed by the GAA/TTC triplet repeat associated with Friedreich's ataxia J Mol Biol 1999 285 2035 2052 10.1006/jmbi.1998.2435 9925783

[B15] Potaman VN Oussatcheva EA Lyubchenko YL Shlyakhtenko LS Bidichandani SI Ashizawa T Sinden RR Length-dependent structure formation in Friedreich ataxia (GAA)n*(TTC)n repeats at neutral pH Nucleic Acids Res 2004 32 1224 1231 10.1093/nar/gkh274 14978261 PMC373408

[B16] Sakamoto N Chastain PD Parniewski P Ohshima K Pandolfo M Griffith JD Wells RD Sticky DNA: self-association properties of long GAA.TTC repeats in R.R.Y triplex structures from Friedreich's ataxia Mol Cell 1999 3 465 475 10.1016/S1097-2765(00)80474-8 10230399

[B17] Kohwi Y Kohwi-Shigematsu T Altered gene expression correlates with DNA structure Genes Dev 1991 5 2547 2554 10.1101/gad.5.12b.2547 1752443

[B18] Sakamoto N Ohshima K Montermini L Pandolfo M Wells RD Sticky DNA, a self-associated complex formed at long GAA*TTC repeats in intron 1 of the frataxin gene, inhibits transcription J Biol Chem 2001 276 27171 27177 10.1074/jbc.M101879200 11340071

[B19] Burnett R Melander C Puckett JW Son LS Wells RD Dervan PB Gottesfeld JM DNA sequence-specific polyamides alleviate transcription inhibition associated with long GAA.TTC repeats in Friedreich's ataxia Proc Natl Acad Sci USA 2006 103 11497 11502 10.1073/pnas.0604939103 16857735 PMC1544198

[B20] Grabczyk E Mancuso M Sammarco MC A persistent RNA.DNA hybrid formed by transcription of the Friedreich ataxia triplet repeat in live bacteria, and by T7 RNAP *in vitro* Nucleic Acids Res 2007 35 5351 5359 10.1093/nar/gkm589 17693431 PMC2018641

[B21] Bentin T Cherny D Larsen HJ Nielsen PE Transcription arrest caused by long nascent RNA chains Biochim Biophys Acta 2005 1727 97 105 15716026 10.1016/j.bbaexp.2004.12.006

[B22] Tous C Aguilera A Impairment of transcription elongation by R-loops *in vitro* Biochem Biophys Res Commun 2007 360 428 432 10.1016/j.bbrc.2007.06.098 17603014

[B23] Roy D Zhang Z Lu Z Hsieh CL Lieber MR Competition between the RNA transcript and the nontemplate DNA strand during R-loop formation *in vitro*: a nick can serve as a strong R-loop initiation site Mol Cell Biol 2010 30 146 159 10.1128/MCB.00897-09 19841062 PMC2798282

[B24] Reddy K Tam M Bowater RP Barber M Tomlinson M Nichol Edamura K Wang YH Pearson CE Determinants of R-loop formation at convergent bidirectionally transcribed trinucleotide repeats Nucleic Acids Res 2011 39 1749 1762 10.1093/nar/gkq935 21051337 PMC3061079

[B25] De Biase I Chutake YK Rindler PM Bidichandani SI Epigenetic silencing in Friedreich ataxia is associated with depletion of CTCF (CCCTC-binding factor) and antisense transcription PLoS One 2009 4 e7914 10.1371/journal.pone.0007914 19956589 PMC2780319

[B26] Yu K Chedin F Hsieh CL Wilson TE Lieber MR R-loops at immunoglobulin class switch regions in the chromosomes of stimulated B cells Nat Immunol 2003 4 442 451 12679812 10.1038/ni919

[B27] Ku S Soragni E Campau E Thomas EA Altun G Laurent LC Loring JF Napierala M Gottesfeld JM Friedreich's ataxia induced pluripotent stem cells model intergenerational GAATTC triplet repeat instability Cell Stem Cell 2010 7 631 637 10.1016/j.stem.2010.09.014 21040903 PMC2987635

[B28] Bidichandani SI Ashizawa T Patel PI The GAA triplet-repeat expansion in Friedreich ataxia interferes with transcription and may be associated with an unusual DNA structure Am J Hum Genet 1998 62 111 121 10.1086/301680 9443873 PMC1376805

[B29] Krasilnikova MM Kireeva ML Petrovic V Knijnikova N Kashlev M Mirkin SM Effects of Friedreich's ataxia (GAA)n*(TTC)n repeats on RNA synthesis and stability Nucleic Acids Res 2007 35 1075 1084 10.1093/nar/gkl1140 17264130 PMC1851639

[B30] Baralle M Pastor T Bussani E Pagani F Influence of Friedreich ataxia GAA noncoding repeat expansions on pre-mRNA processing Am J Hum Genet 2008 83 77 88 10.1016/j.ajhg.2008.06.018 18597733 PMC2443835

[B31] Krasilnikova MM Mirkin SM Replication stalling at Friedreich's ataxia (GAA)n repeats *in vivo* Mol Cell Biol 2004 24 2286 2295 10.1128/MCB.24.6.2286-2295.2004 14993268 PMC355872

[B32] Ohshima K Montermini L Wells RD Pandolfo M Inhibitory effects of expanded GAA.TTC triplet repeats from intron I of the Friedreich ataxia gene on transcription and replication *in vivo* J Biol Chem 1998 273 14588 14595 10.1074/jbc.273.23.14588 9603975

[B33] Al-Mahdawi S Pinto RM Ismail O Varshney D Lymperi S Sandi C Trabzuni D Pook M The Friedreich ataxia GAA repeat expansion mutation induces comparable epigenetic changes in human and transgenic mouse brain and heart tissues Hum Mol Genet 2008 17 735 746 18045775 10.1093/hmg/ddm346

[B34] Rai M Soragni E Jenssen K Burnett R Herman D Coppola G Geschwind DH Gottesfeld JM Pandolfo M HDAC inhibitors correct frataxin deficiency in a Friedreich ataxia mouse model PLoS One 2008 3 e1958 10.1371/journal.pone.0001958 18463734 PMC2373517

[B35] Soragni E Herman D Dent SY Gottesfeld JM Wells RD Napierala M Long intronic GAA*TTC repeats induce epigenetic changes and reporter gene silencing in a molecular model of Friedreich ataxia Nucleic Acids Res 2008 36 6056 6065 10.1093/nar/gkn604 18820300 PMC2577344

[B36] Shishkin AA Voineagu I Matera R Cherng N Chernet BT Krasilnikova MM Narayanan V Lobachev KS Mirkin SM Large-scale expansions of Friedreich's ataxia GAA repeats in yeast Mol Cell 2009 35 82 92 10.1016/j.molcel.2009.06.017 19595718 PMC2722067

[B37] Klinz FJ Gallwitz D Size and position of intervening sequences are critical for the splicing efficiency of pre-mRNA in the yeast Saccharomyces cerevisiae Nucleic Acids Res 1985 13 3791 3804 10.1093/nar/13.11.3791 3892483 PMC341278

[B38] Oberle I Rousseau F Heitz D Kretz C Devys D Hanauer A Boue J Bertheas M Mandel J Instability of a 550-base pair DNA segment and abnormal methylation in fragile X syndrome Science 1991 252 1097 1102 10.1126/science.252.5009.1097 2031184

[B39] Bestor TH Gene silencing. Methylation meets acetylation Nature 1998 393 311 312 10.1038/30613 9620794

[B40] Chen X Mariappan SV Catasti P Ratliff R Moyzis RK Laayoun A Smith SS Bradbury EM Gupta G Hairpins are formed by the single DNA strands of the fragile X triplet repeats: structure and biological implications Proc Natl Acad Sci USA 1995 92 5199 5203 10.1073/pnas.92.11.5199 7761473 PMC41876

[B41] Jones PL Wolffe AP Relationships between chromatin organization and DNA methylation in determining gene expression Semin Cancer Biol 1999 9 339 347 10.1006/scbi.1999.0134 10547342

[B42] Kass SU Pruss D Wolffe AP How does DNA methylation repress transcription? Trends Genet 1997 13 444 449 10.1016/S0168-9525(97)01268-7 9385841

[B43] Razin A CpG methylation, chromatin structure and gene silencing-a three-way connection EMBO J 1998 17 4905 4908 10.1093/emboj/17.17.4905 9724627 PMC1170819

[B44] Eiges R Urbach A Malcov M Frumkin T Schwartz T Amit A Yaron Y Eden A Yanuka O Benvenisty N Ben-Yosef D Developmental study of fragile X syndrome using human embryonic stem cells derived from preimplantation genetically diagnosed embryos Cell Stem Cell 2007 1 568 577 10.1016/j.stem.2007.09.001 18371394

[B45] Tabolacci E Moscato U Zalfa F Bagni C Chiurazzi P Neri G Epigenetic analysis reveals a euchromatic configuration in the FMR1 unmethylated full mutations Eur J Hum Genet 2008 16 1487 1498 10.1038/ejhg.2008.130 18628788

[B46] Cho DH Thienes CP Mahoney SE Analau E Filippova GN Tapscott SJ Antisense transcription and heterochromatin at the DM1 CTG repeats are constrained by CTCF Mol Cell 2005 20 483 489 10.1016/j.molcel.2005.09.002 16285929

[B47] Saveliev A Everett C Sharpe T Webster Z Festenstein R DNA triplet repeats mediate heterochromatin-protein-1-sensitive variegated gene silencing Nature 2003 422 909 913 10.1038/nature01596 12712207

[B48] Greene E Mahishi L Entezam A Kumari D Usdin K Repeat-induced epigenetic changes in intron 1 of the frataxin gene and its consequences in Friedreich ataxia Nucleic Acids Res 2007 35 3383 3390 10.1093/nar/gkm271 17478498 PMC1904289

[B49] Castaldo I Pinelli M Monticelli A Acquaviva F Giacchetti M Filla A Sacchetti S Keller S Avvedimento VE Chiariotti L Cocozza S DNA methylation in intron 1 of the frataxin gene is related to GAA repeat length and age of onset in Friedreich ataxia patients J Med Genet 2008 45 808 812 10.1136/jmg.2008.058594 18697824

[B50] Herman D Jenssen K Burnett R Soragni E Perlman SL Gottesfeld JM Histone deacetylase inhibitors reverse gene silencing in Friedreich's ataxia Nat Chem Biol 2006 2 551 558 16921367 10.1038/nchembio815

[B51] Kim E Napierala M Dent SY Hyperexpansion of GAA repeats affects post-initiation steps of FXN transcription in Friedreich's ataxia Nucleic Acids Res 2011 39 8366 8377 10.1093/nar/gkr542 21745819 PMC3201871

[B52] Kumari D Biacsi RE Usdin K Repeat expansion affects both transcription initiation and elongation in Friedreich ataxia cells J Biol Chem 2011 286 4209 4215 10.1074/jbc.M110.194035 21127046 PMC3039332

[B53] Kumari D Usdin K The distribution of repressive histone modifications on silenced FMR1 alleles provides clues to the mechanism of gene silencing in fragile X syndrome Hum Mol Genet 2010 19 4634 4642 10.1093/hmg/ddq394 20843831 PMC2972696

[B54] Grewal SI Moazed D Heterochromatin and epigenetic control of gene expression Science 2003 301 798 802 10.1126/science.1086887 12907790

[B55] O'Hagan HM Mohammad HP Baylin SB Double strand breaks can initiate gene silencing and SIRT1-dependent onset of DNA methylation in an exogenous promoter CpG island PLoS Genet 2008 4 e1000155 10.1371/journal.pgen.1000155 18704159 PMC2491723

[B56] Matzke M Aufsatz W Kanno T Daxinger L Papp I Mette MF Matzke AJ Genetic analysis of RNA-mediated transcriptional gene silencing Biochim Biophys Acta 2004 1677 129 141 15020054 10.1016/j.bbaexp.2003.10.015

[B57] Shanbhag NM Rafalska-Metcalf IU Balane-Bolivar C Janicki SM Greenberg RA ATM-dependent chromatin changes silence transcription in cis to DNA double-strand breaks Cell 2010 141 970 981 10.1016/j.cell.2010.04.038 20550933 PMC2920610

[B58] Buzas DM Robertson M Finnegan EJ Helliwell CA Transcription-dependence of histone H3 lysine 27 trimethylation at the *Arabidopsis *polycomb target gene FLC Plant J 2011 65 872 881 10.1111/j.1365-313X.2010.04471.x 21276103

[B59] Rinn JL Kertesz M Wang JK Squazzo SL Xu X Brugmann SA Goodnough LH Helms JA Farnham PJ Segal E Chang HY Functional demarcation of active and silent chromatin domains in human HOX loci by noncoding RNAs Cell 2007 129 1311 1323 10.1016/j.cell.2007.05.022 17604720 PMC2084369

[B60] Schmitz KM Mayer C Postepska A Grummt I Interaction of noncoding RNA with the rDNA promoter mediates recruitment of DNMT3b and silencing of rRNA genes Genes Dev 2010 24 2264 2269 10.1101/gad.590910 20952535 PMC2956204

[B61] Heidenfelder BL Makhov AM Topal MD Hairpin formation in Friedreich's ataxia triplet repeat expansion J Biol Chem 2003 278 2425 2431 10.1074/jbc.M210643200 12441336

[B62] Handa V Saha T Usdin K The fragile X syndrome repeats form RNA hairpins that do not activate the interferon-inducible protein kinase, PKR, but are cut by Dicer Nucleic Acids Res 2003 31 6243 6248 10.1093/nar/gkg818 14576312 PMC275460

[B63] Krol J Fiszer A Mykowska A Sobczak K de Mezer M Krzyzosiak WJ Ribonuclease dicer cleaves triplet repeat hairpins into shorter repeats that silence specific targets Mol Cell 2007 25 575 586 10.1016/j.molcel.2007.01.031 17317629

[B64] Lorincz MC Dickerson DR Schmitt M Groudine M Intragenic DNA methylation alters chromatin structure and elongation efficiency in mammalian cells Nat Struct Mol Biol 2004 11 1068 1075 10.1038/nsmb840 15467727

[B65] Xu C Soragni E Chou CJ Herman D Plasterer HL Rusche JR Gottesfeld JM Chemical probes identify a role for histone deacetylase 3 in Friedreich's ataxia gene silencing Chem Biol 2009 16 980 989 10.1016/j.chembiol.2009.07.010 19778726 PMC2909763

[B66] Komarnitsky P Cho EJ Buratowski S Different phosphorylated forms of RNA polymerase II and associated mRNA processing factors during transcription Genes Dev 2000 14 2452 2460 10.1101/gad.824700 11018013 PMC316976

[B67] Guenther MG Levine SS Boyer LA Jaenisch R Young RA A chromatin landmark and transcription initiation at most promoters in human cells Cell 2007 130 77 88 10.1016/j.cell.2007.05.042 17632057 PMC3200295

[B68] Ng HH Robert F Young RA Struhl K Targeted recruitment of Set1 histone methylase by elongating Pol II provides a localized mark and memory of recent transcriptional activity Mol Cell 2003 11 709 719 10.1016/S1097-2765(03)00092-3 12667453

[B69] Vermeulen M Mulder KW Denissov S Pijnappel WW van Schaik FM Varier RA Baltissen MP Stunnenberg HG Mann M Timmers HT Selective anchoring of TFIID to nucleosomes by trimethylation of histone H3 lysine 4 Cell 2007 131 58 69 10.1016/j.cell.2007.08.016 17884155

[B70] Wang P Lin C Smith ER Guo H Sanderson BW Wu M Gogol M Alexander T Seidel C Wiedemann LM Ge K Krumlauf R Shilatifard A Global analysis of H3K4 methylation defines MLL family member targets and points to a role for MLL1-mediated H3K4 methylation in the regulation of transcriptional initiation by RNA polymerase II Mol Cell Biol 2009 29 6074 6085 10.1128/MCB.00924-09 19703992 PMC2772563

[B71] Kubicek S O'Sullivan RJ August EM Hickey ER Zhang Q Teodoro ML Rea S Mechtler K Kowalski JA Homon CA Kelly TA Jenuwein T Reversal of H3K9me2 by a small-molecule inhibitor for the G9a histone methyltransferase Mol Cell 2007 25 473 481 10.1016/j.molcel.2007.01.017 17289593

[B72] Link PA Gangisetty O James SR Woloszynska-Read A Tachibana M Shinkai Y Karpf AR Distinct roles for histone methyltransferases G9a and GLP in cancer germ-line antigen gene regulation in human cancer cells and murine embryonic stem cells Mol Cancer Res 2009 7 851 862 10.1158/1541-7786.MCR-08-0497 19531572 PMC2836864

[B73] Li K Singh A Crooks DR Dai X Cong Z Pan L Ha D Rouault TA Expression of human frataxin is regulated by transcription factors SRF and TFAP2 PLoS One 2010 5 e12286 10.1371/journal.pone.0012286 20808827 PMC2924884

[B74] Chou SH Chin KH Wang AH Unusual DNA duplex and hairpin motifs Nucleic Acids Res 2003 31 2461 2474 10.1093/nar/gkg367 12736295 PMC156048

[B75] LeProust EM Pearson CE Sinden RR Gao X Unexpected formation of parallel duplex in GAA and TTC trinucleotide repeats of Friedreich's ataxia J Mol Biol 2000 302 1063 1080 10.1006/jmbi.2000.4073 11183775

[B76] Roberts RW Crothers DM Stability and properties of double and triple helices: dramatic effects of RNA or DNA backbone composition Science 1992 258 1463 1466 10.1126/science.1279808 1279808

[B77] Zhu J Mayeda A Krainer AR Exon identity established through differential antagonism between exonic splicing silencer-bound hnRNP A1 and enhancer-bound SR proteins Mol Cell 2001 8 1351 1361 10.1016/S1097-2765(01)00409-9 11779509

[B78] Damgaard CK Tange TO Kjems J hnRNP A1 controls HIV-1 mRNA splicing through cooperative binding to intron and exon splicing silencers in the context of a conserved secondary structure RNA 2002 8 1401 1415 10.1017/S1355838202023075 12458794 PMC1370347

[B79] Zuo P Manley JL The human splicing factor ASF/SF2 can specifically recognize pre-mRNA 5' splice sites Proc Natl Acad Sci USA 1994 91 3363 3367 10.1073/pnas.91.8.3363 7512732 PMC43577

